# Open-source cardiac magnetic resonance fingerprinting

**DOI:** 10.1007/s10334-025-01269-9

**Published:** 2025-06-21

**Authors:** Patrick Schuenke, Catarina Redshaw Kranich, Max Lutz, Jakob Schattenfroh, Matthias Anders, Philine Reisdorf, Jeanette Schulz-Menger, Ingolf Sack, Jesse Hamilton, Nicole Seiberlich, Christoph Kolbitsch

**Affiliations:** 1 Physikalisch-Technische Bundesanstalt (PTB), Braunschweig and Berlin, Germany; 2 Department of Radiology, Charité-Universitätsmedizin Berlin, Berlin, Germany; 3 Department of Pediatric Radiology, Charité-Universitätsmedizin Berlin, Berlin, Germany; 4 Working Group on CMR, Experimental and Clinical Research Center, a cooperation between the Max Delbrück Center for Molecular Medicine in the Helmholtz Association and Charité-Universitätsmedizin Berlin, Berlin, Germany; 5 Charité-Universitätsmedizin Berlin, Corporate Member of Freie Universität Berlin and Humboldt-Universität zu Berlin, Berlin, Germany; 6 DZHK (German Centre for Cardiovascular Research), Partner Site Berlin, Berlin, Germany; 7 Department of Radiology, University of Michigan, Ann Arbor, MI USA

**Keywords:** MRI, QMRI, Fingerprinting, MRF, Open-source, Pulseq

## Abstract

**Purpose:**

Cardiac magnetic resonance fingerprinting (cMRF) is a powerful quantitative imaging technique that provides multi-parametric diagnostic information. Here, we introduce an open-source framework for cardiac MRF including open-source pulse sequences, image reconstruction, and parameter estimation tools that are needed for the processing of the data.

**Methods:**

A 2D cMRF sequence with a variable-density spiral readout is implemented using the open-source and vendor-agnostic sequence format Pulseq. Cardiac triggering is used to synchronize acquisition with the rest period of the heart. $T_1$ inversion and $T_2$ preparation pulses are added to ensure accurate parameter estimation. Data acquisition is carried out over 15 heartbeats. The images showing the signal changes over time are reconstructed and matched to a pre-calculated signal dictionary. In addition to the cMRF sequence, spin-echo reference sequences for quality control in phantoms are provided. The method is evaluated in phantom experiments using a T1MES phantom on four different scanners. In vivo experiments were performed to compare the open-source cMRF sequence with a vendor-specific cMRF sequence and clinical sequences used for $T_1$ and $T_2$ mapping of the heart. Three volunteers were imaged on two different scanners.

**Results:**

The error of $T_1$ and $T_2$ over all tissue types present in the T1MES phantom was comparable between all four scanners and on average 4.50 ± 2.48%. $T_1$ and $T_2$ maps obtained in vivo were comparable between the open-source and vendor-specific implementation of cMRF.

**Conclusion:**

The proposed open-source cMRF implementation enables accurate parameter estimation across multiple different scanners. Sequence files, image reconstruction, and parameter estimation scripts are available for reproducible quantitative MRI.

**Supplementary Information:**

The online version contains supplementary material available at 10.1007/s10334-025-01269-9.

## Introduction

Cardiac magnetic resonance imaging (MRI) provides a wide range of different diagnostic information and is for many clinical questions the first line imaging technique [1, 2]. Quantitative mapping of MR relaxation times (e.g., $T_1$ and $T_2$) is of high clinical interest because these maps provide unique information for a wide range of different clinical questions [3–6]. In diseases such as amyloidosis and myocarditis, mapping has been shown to be beneficial for accurate diagnosis [7–9].

One of the main challenges of quantitative mapping is the long acquisition time compared to standard qualitative imaging. Magnetic resonance fingerprinting (MRF) has been proposed to shorten scan times [10]. In addition to obtaining parametric maps efficiently, MRF also yields maps of different complementary diagnostic parameters (e.g., $T_1$, $T_2$ or fat-water fraction), which are intrinsically co-registered to each other. This allows for a multi-parametric assessment on a single voxel basis.

Cardiac MRF (cMRF) has adapted these ideas for multi-parametric assessment of the myocardium [11]. Here, the fact that cMRF provides co-registered parametric maps in a single breath-hold is of special interest because commonly different parameters are acquired in different scans, i.e., different breath-holds, making a voxel-based assessment of different parameters challenging. To enable accurate $T_1$ and $T_2$ mapping of the myocardium, cMRF uses RF pulses with varying flip angles along with inversion pulses and $T_2$-preparation pulses to sensitize the cMRF signal to $T_1$ and $T_2$ relaxation times. Data acquisition is carried out using a spiral or radial readout with high undersampling factors to ensure incoherence of undersampling artifacts over time. Cardiac triggering is commonly used to synchronize data acquisition with cardiac rest period during diastole, although free-running sequences have also been proposed [12, 13]. From the acquired data, dynamic images showing the signal variations over time are reconstructed. The signal curves from a precalculated dictionary are then matched on a voxel-by-voxel basis to the images. More advanced approaches using iterative reconstructions, low-rank approaches and deep-learning have been proposed [11, 14, 15].

One of the primary challenges for the widespread adoption of cMRF lies in its complexity. The method requires specialized pulse sequences and non-Cartesian acquisition trajectories that are not readily accessible. Implementing cMRF on vendor-specific platforms requires advanced expertise in MR sequence programming for each particular vendor. Furthermore, replicating the exact cMRF sequence across different software versions of the same vendor might be difficult, as modules including the $T_2$-preparation pulses are frequently updated from one version to the next. A recent study has also shown that changes to the raw data processing pipeline of a vendor can strongly impact the accuracy of the obtained quantitative parameters, requiring support from the vendor to solve [16]. Other studies on classic mapping approaches have shown that open-source sequences and parameter estimation can improve the reproducibility of quantitative MRI [17].

Here, we present an open-source cMRF framework to overcome these challenges and allow for easy and reproducible cMRF. The framework includes an implementation of a cMRF sequence in the open-source sequence language Pulseq [18], inspired by the sequence presented in Ref. [11]. In addition, Pulseq sequences describing a spin echo sequence with different inversion times and a spin echo sequence with different echo times are provided. These sequences are commonly used as reference sequences to estimate $T_1$ and $T_2$ [19] and provide important quality control for quantitative imaging. Finally, all the image reconstruction code for the cMRF as well as the reference sequences is provided. The proposed open-source cMRF sequence is evaluated in phantoms across four different scanners and compared to a vendor-specific implementation in phantom and in vivo experiments.

## Methods

In the following, we give an overview of the implementation of the proposed open-source cMRF sequence and the reference spin-echo sequences. Image reconstruction and parameter estimation are explained and the experiments and analysis are described.

### Pulseq cMRF sequence

**Fig. 1:**
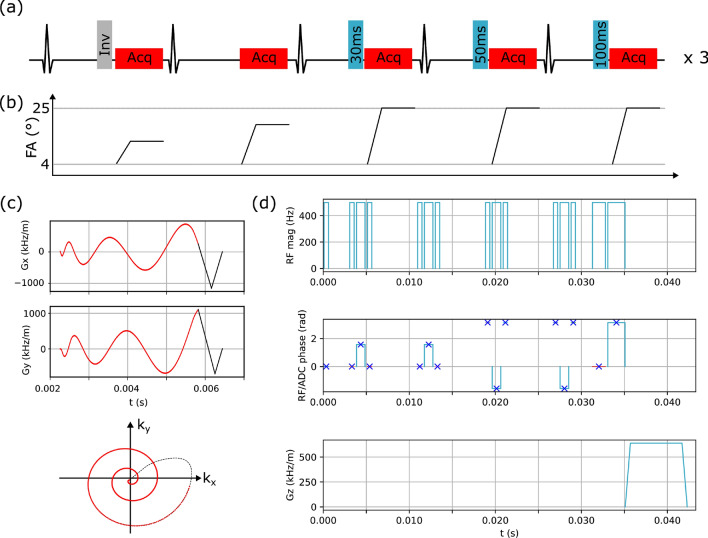
Overview of the cMRF sequence. **a** In the first RR-interval an inversion pulse is applied, in the next RR-interval no preparation pulse is applied, and in the subsequent three RR-intervals $T_2$-preparation pulses with different echo times are applied (e.g., 30 ms, 50 ms and 100 ms). This five heartbeat pattern is repeated three times, leading to a total scan duration of 15 RR-intervals. **b** The flip angle (FA) is varied during the data acquisition between $4^\circ$ and $25^\circ$. **c** Data acquisition is carried out with a variable density spiral trajectory. Data is sampled during the spiral-out part of the trajectory (red) which is followed by a gradient rewinder (black). **d** The MLEV-4 type $T_2$-preparation blocks consist of a (90x, 180y, 180y, −180y, −180y, 270x, −360x) pulse pattern, where the ±180y pulses are realized using (±90x, ±180y, ±90x) composite pulses [20]. The $T_2$-preparation block shown here corresponds to an echo time of 30 ms

The design of the open-source cMRF sequence follows the design presented in Ref. [11]. An overview of the sequence is given in Fig. 1. 2D data acquisition is carried out over 15 heartbeats in a pre-defined mid-diastolic phase of the cardiac cycle. In three cardiac cycles, the data acquisition is preceded by an adiabatic inversion pulse. In nine cardiac cycles, a $T_2$-preparation pulse is applied before the data acquisition with three different echo times. Data acquisition is carried out with a variable density spiral.

The code to generate the variable density spiral trajectories is based on the MATLAB code of Brian Hargreaves [21]. It was translated into Python and adapted to provide a suitable interface for application in PyPulseq [22]. The code allows k-space spacing (1/FOV) to be varied as a function of the k-space radius to increase the sampling efficiency by undersampling the outer portions of k-space. The maximum allowed gradient amplitude and slew rate values can directly be extracted from PyPulseq system limitation objects and are taken into account during the trajectory generation. To calculate the most accurate and time-efficient rewinder gradients while accounting for the gradient raster time, the PyPulseq function make_extended_trapezoid_area was used. This function allows the desired start and end amplitudes, as well as the desired gradient area (0th gradient moment), to be specified and returns the shortest possible extended trapezoid gradient that fulfills these parameters.

The spiral arms were rotated with respect to each other by an approximation of the golden angle [23] as suggested by Hamilton et al. [24]. In total, 48 golden angle spiral arms were pre-calculated with a constant angular increment of $2\pi /48$, which served as a dictionary for the available trajectories. The starting angle for the first readout was $0^\circ$. For the following readouts, the starting angle was calculated by adding a golden angle increment. A trajectory was then selected from the trajectory dictionary, which had the starting angle closest to the current starting angle calculated based on the Golden angle increment. As an example, the starting angle for the spiral arm with index 100 is $100 \times 2\pi \times (1 - 2/(1 + \sqrt{5}))$ which is $71^\circ$. Therefore, the trajectory with the starting angle of $67.5^\circ$ was selected for the readout with index 100 which is the closest entry to $71^\circ$.

The MLEV-4 type $T_2$-preparation block shown in Fig. 1 features a ($90x, 180y, 180y, -180y, -180y, 270x, -360x$) flip angle pattern, with the $180^\circ$ refocusing pulses (±180y) implemented via a ($\pm 90x, \pm 180y, \pm 90x$) composite pulse design [20]. This entire $T_2$-preparation block is modular, allowing for adjustments to RF pulse duration and echo time. Similarly, the $T_1$-preparation/inversion block is modular, enabling customization of inversion time, RF pulse duration, and spoiler gradient duration.

To support image reconstruction, relevant sequence details and the spiral trajectory for each acquisition are saved in an ISMRM raw data (*.MRD) file [25], created in parallel with the Pulseq sequence. After data acquisition on any MRI scanner, the raw data can be exported and converted to the MRD format. Using the Python code provided in our GitHub repository, both MRD files can then be merged into a single file containing all data and header information, as well as trajectory information required for image reconstruction.

For the reconstruction of the different qualitative images, we used a sliding window approach [26]. For each dynamic image, multiple spiral arms are combined and spiral arms are shared between neighboring images (sliding window). An extended phase graph (EPG) approach is used to calculate the signal evolution during the cMRF acquisition [27]. The cMRF sequence is triggered to the cardiac cycle and therefore the time between data acquisition and preparation blocks in the sequence depend on the heart rate. A separate dictionary was calculated for each subject. The signal for different $T_1$ and $T_2$ times is estimated for each RF pulse with $T_1$ values selected as [50:10:2000 ms, 2020:20:3000 ms, 3050:50:5000 ms] and $T_2$ values selected as [6:2:100 ms, 105:5:200 ms, 220:20:500 ms]. To take the sliding window reconstruction into account, the signal in each sliding window range is then averaged to obtain the final signal curves. This signal dictionary is then matched to the reconstructed images on a pixel-by-pixel basis. The $T_1$ and $T_2$ times corresponding to the highest dot product between image and dictionary signal curves are selected for the final quantitative maps.

### Reference scans for $T_1$ and $T_2$ mapping

The reference $T_1$ and $T_2$ values were estimated with spin-echo sequences using a Cartesian sampling pattern, which obtain a single readout line after the $90^\circ$–$180^\circ$-RF-pulses. Data are acquired at different time points after an adiabatic inversion pulse for the $T_1$ estimation and with different echo-times for the $T_2$ estimation, respectively. The quantitative maps are obtained using dictionary mapping with a dictionary containing $T_1$ values in [50:10:2000 ms, 2020:20:3000 ms, 3050:50:5000 ms] for the $T_1$ reference scan and $T_2$ values in [6:2:100 ms, 105:5:200 ms, 220:20:500 ms] for the $T_2$ reference scan.

### Experiments

In this study, we carried out three different sets of experiments to evaluate the reproducibility and accuracy of the open-source cMRF sequence across different scanners and to evaluate the sequence in in vivo experiments.

To demonstrate that the proposed open-source cMRF sequence provides accurate $T_1$ and $T_2$ quantification on different scanner platforms, we carried out phantom experiments using a T1MES phantom [19]. We used the same phantom on four 3 T Siemens scanners (Siemens Healthineers, Erlangen, Germany) with different software versions (Table 1).

**Table 1:** MR scanners used for the evaluation of the open-source cMRF sequence

Scanner	1	2	3	4
Model name	Cima.X	Skyra	Lumina	SkyraFit
IDEA version	XA61	XA30	XA20	VE11C
Pulseq version	1.4.2	1.4.2	1.4.2	1.4.2

All scanners were Siemens 3 T scanners (Siemens Healthineers, Erlangen, Germany)

We selected a lower maximum gradient strength and gradient slew rate (leading to longer gradient durations) to what would have been possible on most of the scanners to ensure that the sequence can be run with the exact same settings on each scanner. Values of 30 mT/m for the maximum gradient amplitude and 100 T/m/s for the maximum slew rate were used for all of these phantom experiments. Data acquisition was carried out with a field-of-view (FOV) of 128 $\times$ 128 $\hbox {mm}^2$ with a pixel size of 1 $\hbox {mm}^2$ and a slice thickness of 8 mm. For the open-source cMRF sequence, the echo times of the $T_2$-preparation pulses were selected as 30 ms, 50 ms and 100 ms. An ECG-signal was simulated for a heart rate of 60 bpm. In each cardiac cycle, 47 spiral arms were acquired with 355 sampling points along each spiral arm. The TE and TR were set to 1.6 ms and 10 ms, respectively.

In addition to the open-source cMRF sequence, we also acquired data with the $T_1$ and $T_2$ spin-echo reference sequences. The FOV, voxel size and slice thickness were set to be the same as those used in the cMRF sequence. For the $T_1$ spin-echo reference sequence, data at inversion times of 25 ms, 50 ms, 300 ms, 600 ms, 1200 ms, 2400 ms and 4800 ms were obtained with TE of 20 ms which took 1 h 59 min. For the $T_2$ spin-echo reference sequence, echo times of 24 ms, 50 ms, 100 ms, 200 ms and 400 ms were obtained which took 1 h 25 min. The TR for both sequences was 8 s. Although the same phantom was used for all phantom scans, the $T_1$ and $T_2$ reference sequences were carried out at each scanner to compensate for potential relaxation time variations, such as those caused by temperature differences.

A direct reconstruction was used for image reconstruction, where each image was reconstructed by applying a density compensation function to the spiral k-space data followed by gridding on a Cartesian grid and Fast Fourier transformation. For the Cartesian spin-echo reference scans only the Fast Fourier transformation was needed. The data from the different receiver coils were combined by a weighted sum using coil sensitivity maps.

For all phantom experiments, parameter maps obtained with the open-source cMRF sequence were compared to the $T_1$ and $T_2$ maps obtained with the spin-echo reference sequences for each of the scanners separately. In each tube of the T1MES phantom, a region-of-interest (ROI) was manually delineated. The mean value and standard deviation within this ROI was compared. A linear fit was carried out and the relative root-mean-squared-error (RMSE) over all nine tubes was calculated between the cMRF and the reference sequences. Figures showing $T_1$ and $T_2$ maps follow the recommendation provided by Fuderer et al. [28].

In the second set of experiments, we obtained $T_1$ and $T_2$ maps in a healthy volunteer (male, 34 years). We also obtained $T_1$ and $T_2$ maps with a vendor-specific cMRF sequence. The sequence follows the method outlined in Ref. [11] and is available as a prototype sequence for Siemens scanners. These experiments were only carried out on scanner 1 as the vendor-specific cMRF sequence was only available there. The FOV was 300 $\times$ 300 $\hbox {mm}^2$ with a resolution of 1.56 $\times$ 1.56 $\hbox {mm}^2$ and a slice thickness of 8 mm. We maximized the peak gradient strength (180 mT/m) and gradient slew rate (150 T/m/s) and adapted the sequence parameters of the open-source cMRF sequence as much as possible to the vendor-specific cMRF sequence. $T_2$-preparation pulses with echo times of 30 ms, 50 ms and 80 ms, a TE of 1.6 ms and a TR of 6.6 ms was used. 47 spiral arms were acquired in each cardiac cycle with 355 readout points along a spiral arm. For the phantom scans, a heart rate of 60 bpm was simulated. For the in vivo scan, data acquisition was carried out during mid-diastole. A cine acquisition was carried out to determine the rest-period of the heart during diastole. The parameters of the vendor-specific cMRF sequence were the same, except for TE and TR which were 1 ms and 5.5 ms, respectively. In one cardiac cycle 45 spiral arms were obtained, each with 1388 sampling points. Fat-suppression was used for this sequence.

For the in vivo experiments, the spin-echo reference sequences could not be used due to their long acquisition times. Clinically recommended sequences were used instead [29]. For $T_1$ mapping, a 5(3)3 Modified look-locker inversion recovery (MOLLI) sequence was used [30]. $T_2$ maps were obtained using a $T_2$-prepared single-shot gradient echo sequence (T2prep) [31]. The echo times of the $T_2$-preparation blocks were 0 ms, 35 ms and 55 ms. Both sequences were ECG-triggered with the same trigger time as the cMRF sequences. MOLLI and T2prep are part of a software package for cardiac imaging provided by the vendor. $T_1$ and $T_2$ estimation was carried out on the scanner using the vendor software. The accuracy of sequences used for in vivo imaging was also evaluated in phantom experiments by comparing the results to the spin echo reference sequences. In the following we refer to the MOLLI and T2prep sequence as clinical sequences.

In the third set of experiments, we carried out a traveling volunteer study, where we obtained $T_1$ and $T_2$ maps with the open-source cMRF sequence in three volunteers on two different scanners. The scans were conducted on two successive days.

All in vivo scans of the second and third set of experiments were carried out on a short-axis orientation and each scan was obtained in a single breath-hold. These in vivo experiments were approved by the ethic committee of the responsible institution and carried out in accordance with relevant guidelines and regulations. The subjects gave written informed consent to participate in this study.

For the in vivo scans, each image was reconstructed using an iterative SENSE approach due to the larger FOV and hence higher level of undersampling artifacts [32]. The coil sensitivity maps needed for both reconstructions were calculated from the average image utilizing all acquired data with the method proposed by Inati et al. [33]. Image reconstructions and parameter estimations for the open-source cMRF sequence were performed offline using MRpro [34], a PyTorch-based MR image reconstruction and processing package. The processing time for a single cMRF in vivo acquisition took less than 2 min (50 s for iterative image reconstruction, 50 s for dictionary calculation, 1 s for dictionary matching) on a PC with 12 CPUs and 192 GB RAM without GPU acceleration.

## Results

**Fig. 2:**
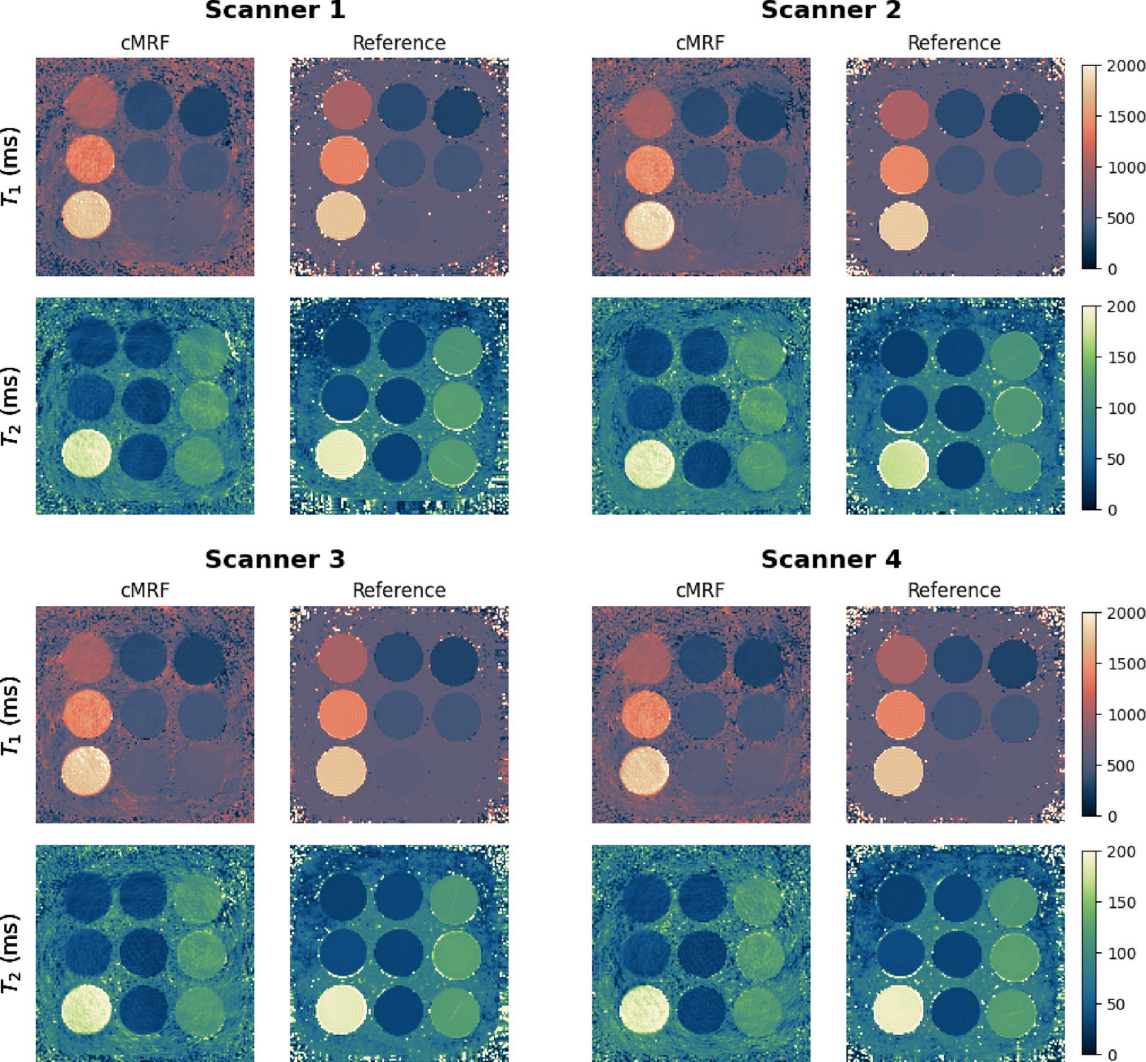
Phantom experiments performed on four different scanners. $T_1$ and $T_2$ maps obtained with the proposed open-source cMRF sequence (cMRF) and spin-echo reference sequences (reference) are shown

**Fig. 3:**
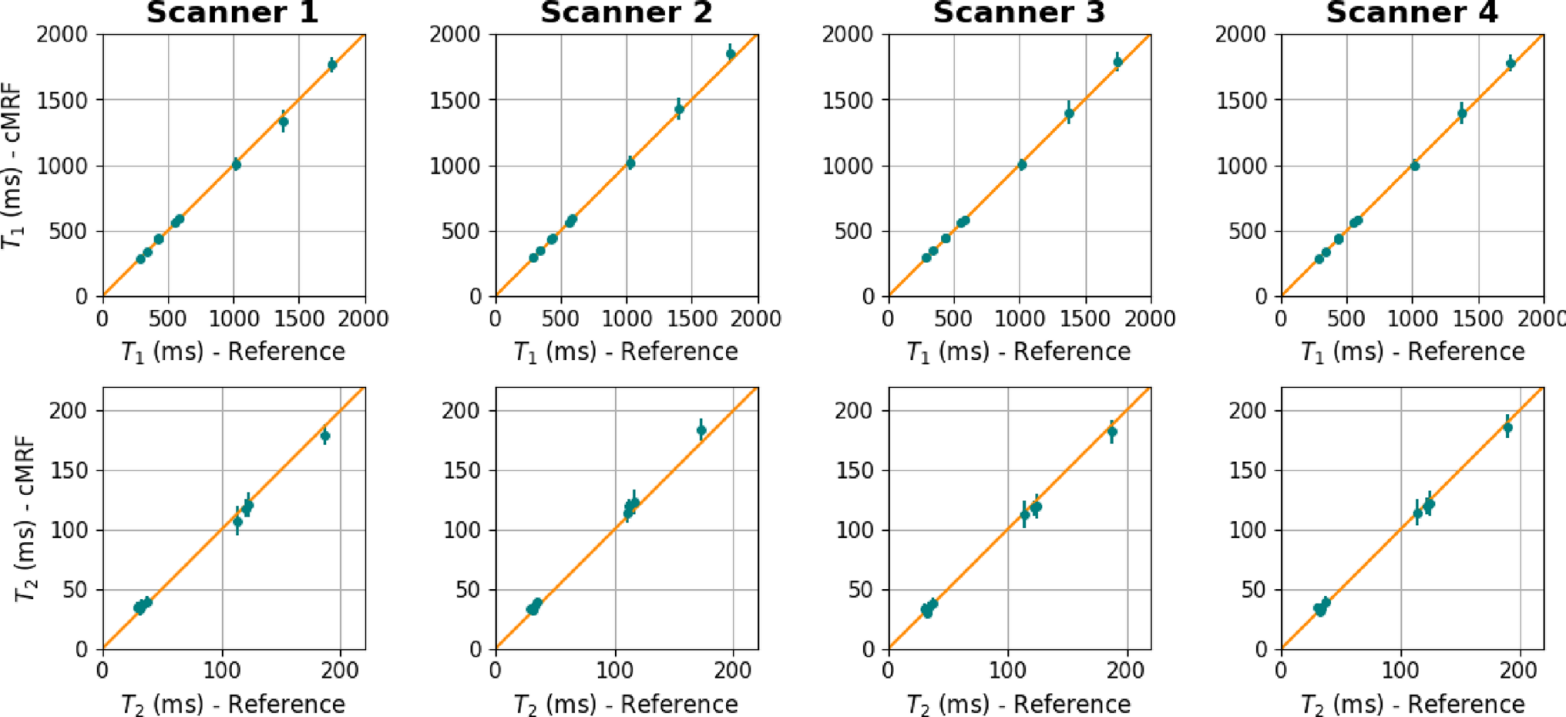
$T_1$ and $T_2$ values estimated within a circular ROI in each tube of the T1MES phantom. For each tube, the average value and the standard deviation as error bars are plotted. The values for all tubes lie close to the identity line (orange) indicating high agreement between the open-source cMRF sequence (cMRF) and the reference sequences (reference)

**Fig. 4:**
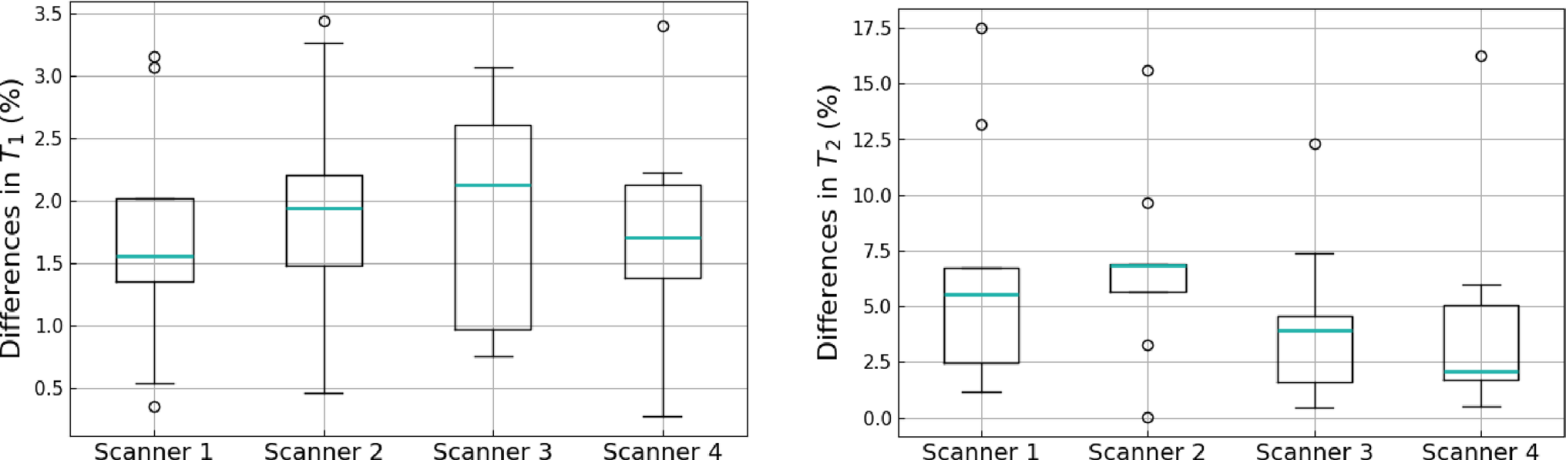
Relative error of $T_1$ and $T_2$ measured in each of the nine tubes of the T1MES phantom between open-source cMRF and reference spin-echo measurements for all four scanners included in this study

Figure 2 shows the $T_1$ and $T_2$ maps obtained from the T1MES phantom with the open-source cMRF sequence and the spin-echo reference sequences for all four scanners. ROI-averaged values for each tube are compared between open-source cMRF and reference sequences in Fig. 3. Relative errors for each tube are shown in Fig. 4. Table 2 shows the slope, intercept and $R^2$ values from the linear fits comparing the $T_1$ and $T_2$ values obtained using the open-source cMRF sequence and the reference sequences. The values for all nine tubes are close to the identity line in Fig. 3, with a slope near 1, a small intercept, and an $R^2$ greater than 0.99. This demonstrates the high accuracy of the quantitative values measured using the open-source cMRF sequence. The RMSE average over all tubes is comparable between all scanners and on average 2.02 ± 0.10% for T1 and 6.97 ± 1.17% for T2, respectively. In addition to the RMSE, which quantifies the accuracy of the method, we calculated the mean standard deviation of the error in each tube as an indicator of precision. This metric also showed comparable behavior across all scanners, with values of 4.25 ± 0.18% for T1 and 10.41 ± 0.77% for T2.

**Table 2:** Parameters (slope and intercept) of a linear fit between the $T_1$ and $T_2$ values obtained with the spin-echo reference sequences and the open-source cMRF sequence for all tine tubes of the T1MES phantom

	Scanner	1	2	3	4
$T_1$	Slope	0.985	1.027	1.023	1.016
	Intercept (ms)	11.112	$-$ 6.821	$-$ 4.856	$-$ 2.237
	$R^{2}$	0.9990	0.9994	0.9994	0.9994
	RMSE ($\%$)	1.93	2.16	2.07	1.93
	Mean relative standard deviation ($\%$)	4.46	4.03	4.27	4.23
$T_2$	Slope	0.934	1.056	0.956	0.971
	Intercept (ms)	4.929	0.438	1.952	1.763
	$R^{2}$	0.9988	0.9991	0.9988	0.9988
	RMSE ($\%$)	8.29	7.93	5.51	6.17
	Mean relative standard deviation ($\%$)	10.98	9.28	10.71	10.68

Corresponding $R^{2}$ and RMSE values are also shown

**Fig. 5:**
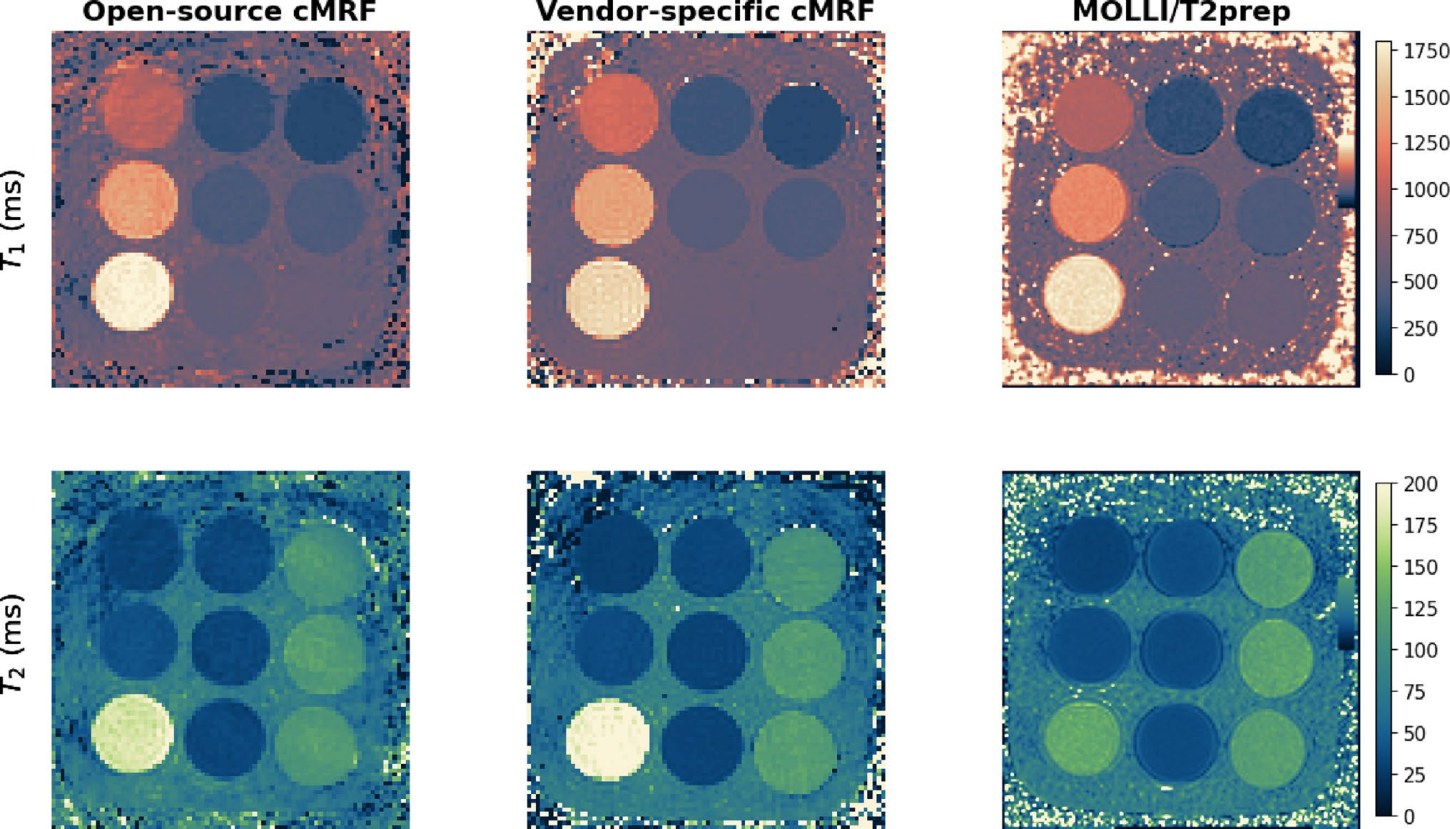
Phantom experiments using the in vivo scan parameters. $T_1$ and $T_2$ maps obtained with the proposed open-source cMRF sequence, the vendor-specific cMRF sequence and the clinical sequences for cardiac $T_1$ and $T_2$ mapping (MOLLI/T2prep). The maps were cropped to focus on the phantom

**Table 3:** Parameters (slope and intercept) of a linear fit between the $T_1$ and $T_2$ values obtained with the spin-echo reference sequence and the open-source cMRF sequence, the vendor-specific cMRF sequence and the clinical sequences for cardiac $T_1$ and $T_2$ mapping (MOLLI/T2prep) for all nine tubes of the T1MES phantom

		Open-source cMRF	Vendor-specific cMRF	MOLLI/T2prep
$T_1$	Slope	1.069	0.994	0.977
	Intercept (ms)	$-$ 33.482	38.880	2.291
	$R^{2}$	0.9987	0.9951	0.9970
	RMSE ($\%$)	3.01	7.08	3.58
$T_2$	Slope	0.935	1.068	0.805
	Intercept (ms)	3.979	$-$ 4.477	11.290
	$R^{2}$	0.9996	0.9924	0.9090
	RMSE ($\%$)	4.39	3.65	11.37

Corresponding $R^{2}$ and RMSE values are also shown

**Fig. 6:**
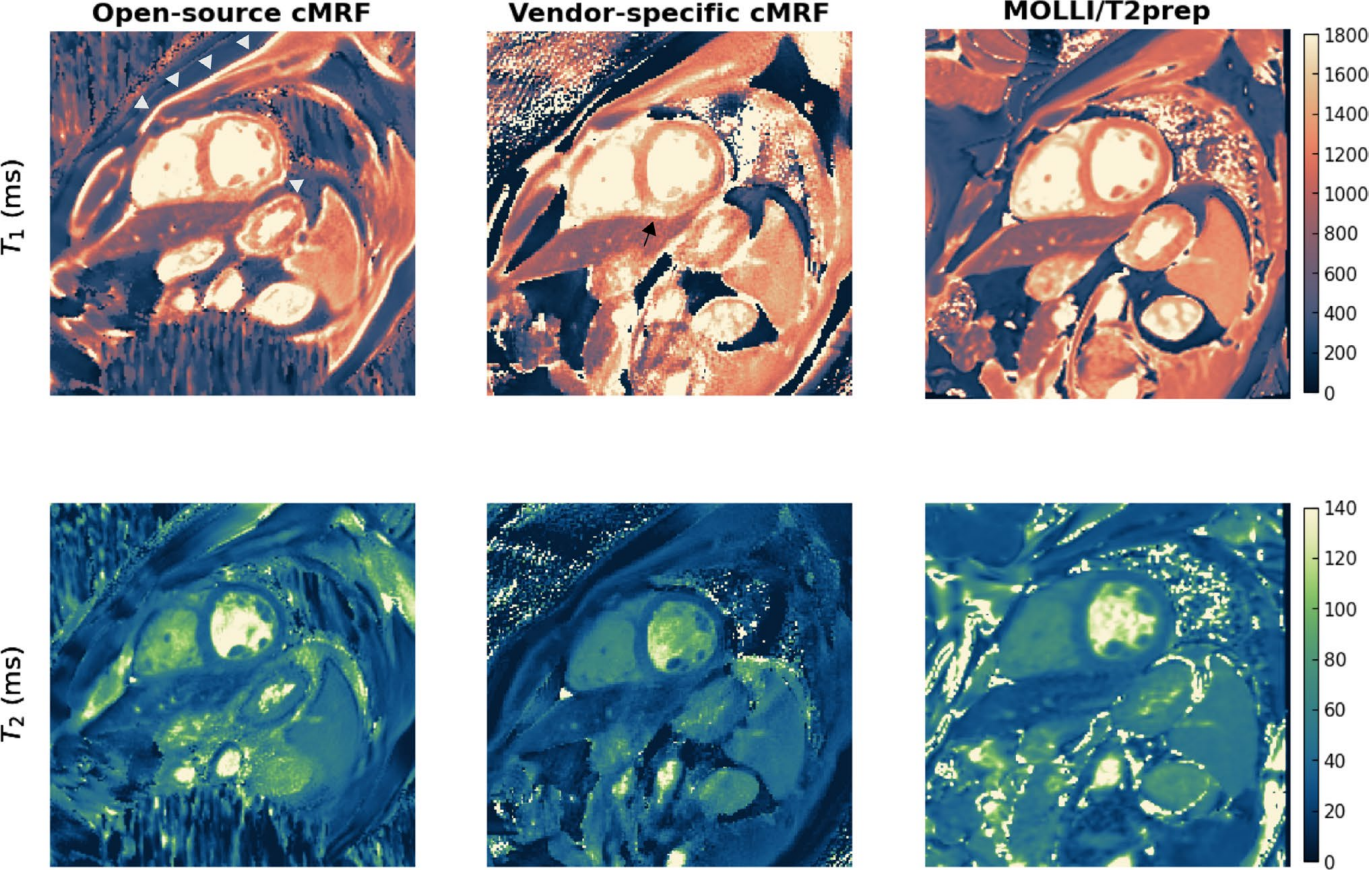
$T_1$ and $T_2$ maps obtained in the in vivo experiments using the proposed open-source cMRF sequence, the vendor-specific cMRF sequence and the clinical sequences for cardiac $T_1$ and $T_2$ mapping (MOLLI/T2prep). The displayed region of the maps obtained with cMRF sequences was adapted to focus on the heart. All methods yield high image quality. The open-source cMRF sequence shows higher fat-water shift in the $T_1$ maps compared to MOLLI (white arrow heads). Increase in $T_1$ can be seen in the vendor-specific cMRF results around the coronary arteries (black arrow heads)

**Table 4:** Results of the traveling volunteer study

	T1 (ms)—scanner 1	T1 (ms)—scanner 3	T2 (ms)—scanner 1	T2 (ms) - Scanner 3
Subject 1	1205.45 ± 49.06	1238.18 ± 28.86	29.27 ± 2.60	36.73 ± 1.29
Subject 2	1204.55 ± 70.76	1208.18 ± 66.03	27.27 ± 2.60	32.91 ± 2.87
Subject 3	1230.00 ± 51.87	1211.18 ± 60.38	30.36 ± 1.15	32.94 ± 3.70

Mean values of $T_1$ and $T_2$ measured within a ROI in the septum for three subjects scanned at two scanners

**Fig. 7:**
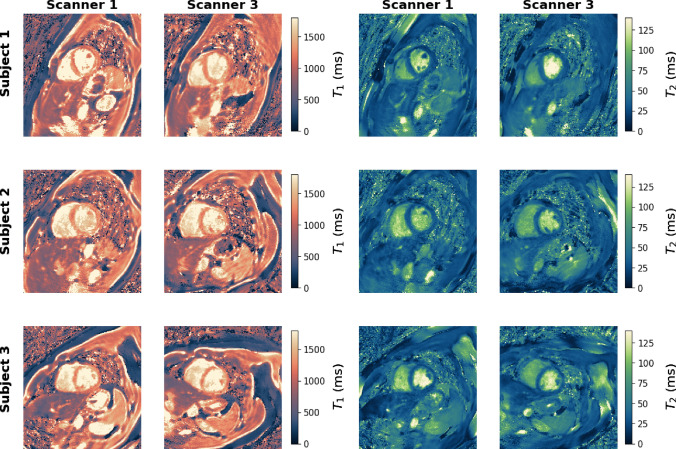
$T_1$ and $T_2$ maps obtained with the open-source cMRF sequence at two different MR scanners in three healthy volunteers

A comparison between the open-source cMRF sequence, vendor-specific cMRF sequence and clinical sequences to the spin-echo reference sequences for the T1MES phantom is shown in Fig. 5. The adapted scan parameters of the open-source cMRF sequence did not have an impact on the accuracy (RMSE) of the $T_1$ and $T_2$ values (Table 3). The vendor-specific cMRF sequence provided $T_1$ and $T_2$ values with similarly high accuracy and had a comparable RMSE value. The clinical sequences MOLLI and $T_2$-prepared gradient echo, respectively, showed higher RMSE values compared to the cMRF sequences. Especially for the $T_2$-prepared gradient echo sequence, the highest $T_2$ value, which is larger than 150 ms, was strongly underestimated leading to a RMSE error of 11.4% for $T_2$ compared to 4.4% for the open-source cMRF sequence. Nevertheless, this sequence is optimized for $T_2$ values in the myocardium, which are in the range of 50 ms and hence this was to be expected. More information can be found in online resource 1.

Figure 6 shows $T_1$ and $T_2$ maps obtained in vivo with the two different cMRF implementations and the clinical sequences. The overall image quality is comparable between all methods with accurate depiction of cardiac features.

The results of the traveling volunteer study are shown in Fig. 7. Comparable $T_1$ and $T_2$ maps could be obtained on two different scanners, which is also confirmed by the quantitative values measured in the septum (Table 4).

## Discussion

The open-source cMRF method presented in this study showed high accuracy for both $T_1$ and $T_2$ mapping with average relative RMSE values of 4.50 ± 2.48% compared to spin-echo reference sequences. The accuracy of the sequence was comparable across four different scanners with different software versions demonstrating high inter-scanner reproducibility. The precision of the open-source cMRF method was also comparable between the different scanners, with an average standard deviation of the error of 7.33 ± 4.4%. Similar accuracy and image quality compared to a vendor-specific cMRF implementation could be achieved.

A major advantage of open-source vendor-independent sequence implementations are their straight-forward adaptability. In this study, we presented an open-source implementation inspired by one specific cMRF implementation proposed by Hamilton et al. [11]. Several other cMRF methods have been presented since then, extending the cMRF method in include e.g., to $T_2^*$ and fat-fraction quantification [15, 35] or 3D imaging [36]. The main principle of advanced data acquisition combined with different preparation pulses is used for most of these techniques, and thus the proposed approach serves as a valuable basis for these methods and can be easily adapted to them. The same applies to measurements at other field strengths, which may require an adjustment of the selected sequence parameters like echo and inversion times of the preparation blocks.

For the phantom experiments, we created the open-source cMRF sequence using hardware limits that allowed to run the same sequence on all systems. This reduced the impact of sequence variations on the final data. Nevertheless, the phantom results obtained with the high-performance settings used for the in vivo scans (Fig. 5) also showed comparable results. This indicates that changes in the sequence due to different hardware limits are well described by the signal model and do not lead to a bias of the results. One exception might be magnetization transfer effects, which are currently not part of the cMRF-EPG model.

A hyperbolic secant pulse was used as the inversion pulse for both the spin-echo reference sequence and the open-source cMRF implementation. Studies have shown that although hyperbolic secant pulses provide homogeneous inversion, a more accurate inversion can be achieved with more advanced pulses [37]. The modular approach of the open-source cMRF sequence would allow a straightforward adaption of the inversion pulse, which could further improve the accuracy.

One challenge of Pulseq is cardiac triggering. Pulseq allows for trigger events to be included in the sequence, but the trigger delay, i.e., the time between R-peak and the start of the acquisition or preparation pulse, must be defined when the sequence file is created. An interactive adaptation via the scanner user interface is currently not possible. In this study, we overcame this challenge by preparing several sequence files with different cardiac trigger times and then selecting the version most suitable for the subject’s heart rate.

Until now, we were only able to collect data on Siemens scanners because ISMRMRD is fully supported only by Siemens. For some other vendors, raw data converters to ISMRMRD exist but support only very specific sequences. Therefore, reproducible reconstruction and parameter estimation were only possible for data acquired on Siemens scanners. However, in general, Pulseq interpreters are currently available at least for Siemens, GE and Philips [18, 38–40].

In the presented implementation of the cMRF sequence, we approximated the golden angle spiral trajectory by a set of uniformly spaced spiral arms as suggested by Hamilton et al. [24]. This was done, on the one hand, to match the vendor-specific implementation of the cMRF sequence. Additionally, this approach significantly reduced the size of the Pulseq file, decreasing the time required to load the sequence on the scanner. Each unique gradient needs to be saved in the Pulseq file, which, in our case, would result in more than 700 different readout gradients. By approximating the trajectory, the Pulseq file contained only the 48 unique spiral arms. In general, spiral trajectories can be very sensitive to inaccuracies of the gradient system and several approaches have been proposed to correct for these gradient inaccuracies [41–43]. The spiral readout in this study (355 samples, 3.55 ms duration) was very short, and no artifacts from gradient inaccuracies were observed.

Figure 6 shows a visible fat-water shift especially in the $T_1$ map for the open-source cMRF implementation. This could likely be resolved using a larger receiver bandwidth, which would also increase the number of samples along each spiral arm, making it more comparable to the vendor-specific cMRF sequence. Although this did not impact the multi-scanner phantom comparison, it should be further investigated to ensure reliable and accurate in vivo scanning. The vendor-specific sequence utilized fat suppression and therefore no artifacts due to the fat-water shift were expected. Such artifacts may also be mitigated by using fat/water separated cMRF sequences such as the rosette sequence used in Liu et al. [44]. Other differences, such as increased $T_1$ values around the coronary arteries (black arrow head in Fig. 6) in the vendor-specific cMRF sequence might also be due to differences in the sequence implementation.

The traveling volunteer study showed good agreement both visually (Fig. 7) and quantitatively (Table 4). Nevertheless, further experiments are necessary as the slice orientation and positioning was different between both scans and hence a direct comparison of both maps is not possible.

The quantitative tissue property parameters were estimated from the acquired data by reconstructing dynamic images followed by the matching with a pre-calculated dictionary. This provided sufficient image quality for this study. However, there are more advanced approaches to estimate cMRF parameters such as low-rank reconstructions [15] or deep-learning based techniques [14].

In the repository associated to this article (https://github.com/PTB-MR/cMRF), we provide the Pulseq sequence files, as well as a Jupyter notebook that demonstrates image reconstruction, dictionary calculation, and parameter estimation for the open-source cMRF sequence and the Pulseq-based spin-echo reference sequences. All raw data files from the phantom scans obtained at the different scanners are provided via zenodo. These files provide a good starting point for other research groups to implement and evaluate more advanced image reconstruction and parameter estimation approaches. We believe that this fully transparent end-to-end open-source MRF framework is an important step towards reproducible and accurate quantitative MRI.

## Conclusion

Cardiac MRF can provide valuable diagnostic information for a range of different clinical questions. In this work, we present an open-source cMRF framework based on Pulseq, which enables accurate and reproducible $T_1$ and $T_2$ quantification over a range of different MR scanners with different software versions. The proposed cMRF sequence was also evaluated in in vivo scans, showing good reproducibility and comparable results to a vendor-specific cMRF sequence. In addition to the necessary scripts to create the open-source cMRF sequence, we also provide Pulseq sequences of spin echo reference scans for the quantification of $T_1$ and $T_2$ and the required scripts for image reconstruction and parameter estimation, enabling reproducible parameter quantification.

## Supplementary Information

Below is the link to the electronic supplementary material.

Supplementary file 1 (pdf 126 KB)

## Data Availability

The Python scripts for generating the Pulseq sequence files for the cMRF sequence, as well as the T1 and T2 spin-echo reference sequences, are publicly available in our GitHub repository: https://github.com/PTB-MR/cMRF. MR raw data (in ISMRM raw data format) supporting Figs. 2, 3 and 4 and Table 2 are accessible via Zenodo at https://zenodo.org/records/14251660 [45]. Data supporting Figs. 5 and Table 3 are available upon request. To protect the volunteers privacy, the data underlying Fig. 6 and Fig. 7 are not publicly available. The script to reproduce Figs. 2 and 3, including image reconstruction, quantitative parameter estimation, and analysis, is included in the GitHub repository: https://github.com/PTB-MR/cMRF.
