# Supplementary material for: Open-source cardiac magnetic resonance fingerprinting
Source: MAGMA. 2025 Jun 21;38(4):665–77. doi: 10.1007/s10334-025-01269-9 (PMC12443904; doi:10.1007/s10334-025-01269-9)
Supplement: Supplementary file 1 — (pdf 126 KB) [file 10334_2025_1269_MOESM1_ESM.pdf]

Magnetic Resonance Materials in Physics, Biology and Medicine

Open-Source Cardiac Magnetic Resonance Fingerprinting

Patrick Schuenke[1,2*], Catarina Redshaw Kranich[1], Max Lutz[1], Jakob Schattenfroh[2], Matthias Anders[3], Philine Reisdorf[4,5,6], Jeanette Schulz-Menger[4,5,6], Ingolf Sack[2], Jesse Hamilton[7], Nicole Seiberlich[7], Christoph Kolbitsch[1]

[1*]Physikalisch-Technische Bundesanstalt (PTB), Braunschweig and Berlin, Germany.

[2]Department of Radiology, Charit ´e – Universit ¨atsmedizin Berlin, Berlin,Germany.

[3]Department of Pediatric Radiology, Charit ´e – Universit ¨atsmedizin Berlin, Berlin,Germany.

[4]Working Group on CMR, Experimental and Clinical Research Center, a cooperation between the Max Delbrück Center for Molecular Medicine in the Helmholtz Association and Charité – Universitätsmedizin Berlin, Berlin, Germany.

[5]Charité – Universitätsmedizin Berlin, coorporate member of Freie Universität Berlin and Humboldt-Universität zu Berlin, Berlin, Germany.

[6]DZHK (German Centre for Cardiovascular Research), partner site Berlin, Berlin, Germany.

[7]Department of Radiology, University of Michigan, Ann Arbor, Michigan, USA

*Corresponding author: Patrick Schuenke, patrick.schuenke@ptb.de

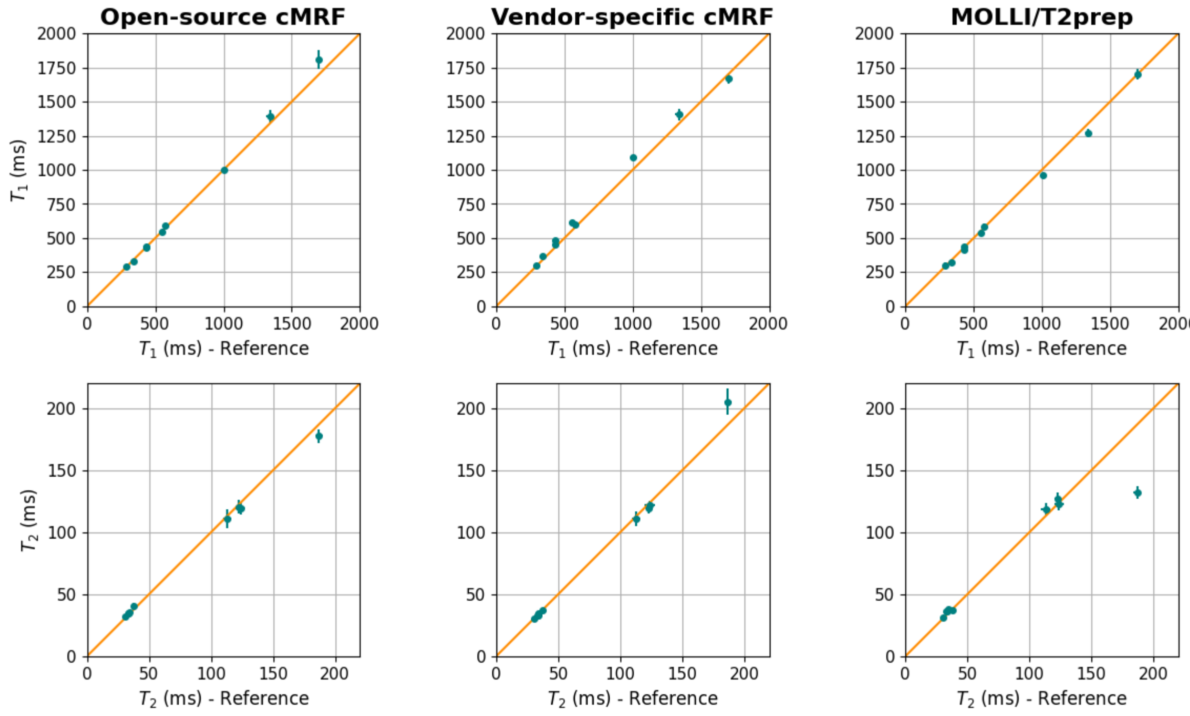

Online resource 1: T_1 and T_2 values estimated within a circular ROI in each tube of the T1MES phantom. For each tube the average value and the standard deviation as error bars are plotted. The values for all tubes lie close to the identity line (orange) indicating high agreement between the open-source cMRF sequence and the vendor-specific cMRF sequence compared to the reference spin-echo sequences (Reference). The clinically used sequence for T_2 mapping (T2prep) shows larger errors for high T_2 values which was to be expected as this sequence is optimised for lower T_2 values.
